# Cardiac Fibrosis: Cellular Effectors, Molecular Pathways, and Exosomal Roles

**DOI:** 10.3389/fcvm.2021.715258

**Published:** 2021-08-16

**Authors:** Wenyang Jiang, Yuyan Xiong, Xiaosong Li, Yuejin Yang

**Affiliations:** ^1^State Key Laboratory of Cardiovascular Disease, National Center for Cardiovascular Diseases, Fuwai Hospital, Chinese Academy of Medical Science and Peking Union Medical College, Beijing, China; ^2^State Key Laboratory of Cardiovascular Disease, Department of Cardiology, National Center for Cardiovascular Diseases, Fuwai Hospital, Chinese Academy of Medical Science and Peking Union Medical College, Beijing, China

**Keywords:** cardiac fibrosis, cellular effectors, mechanisms, exosome, treatment

## Abstract

Cardiac fibrosis, a common pathophysiologic process in most heart diseases, refers to an excess of extracellular matrix (ECM) deposition by cardiac fibroblasts (CFs), which can lead to cardiac dysfunction and heart failure subsequently. Not only CFs but also several other cell types including macrophages and endothelial cells participate in the process of cardiac fibrosis *via* different molecular pathways. Exosomes, ranging in 30–150 nm of size, have been confirmed to play an essential role in cellular communications by their bioactive contents, which are currently a hot area to explore pathobiology and therapeutic strategy in multiple pathophysiologic processes including cardiac fibrosis. Cardioprotective factors such as RNAs and proteins packaged in exosomes make them an excellent cell-free system to improve cardiac function without significant immune response. Emerging evidence indicates that targeting selective molecules in cell-derived exosomes could be appealing therapeutic treatments in cardiac fibrosis. In this review, we summarize the current understandings of cellular effectors, molecular pathways, and exosomal roles in cardiac fibrosis.

## Introduction

Cardiac fibrosis, marked by an excess of extracellular matrix (ECM) deposition by cardiac fibroblasts (CFs), is a common pathophysiologic process in most heart diseases such as myocardial infarction (MI), hypertensive heart disease, and different types of cardiomyopathies (1, 2) and impair the heart physically and electrically. Taking acute MI (AMI) as an example, sudden massive loss of cardiomyocytes triggers an intense inflammation and causes the dead myocardium to be replaced with a collagen-based scar (3), which is critical to prevent cardiac rupture. However, prolonged or excessive fibrotic responses could remarkably lead to excessive ECM deposition, which results in hardening of myocardium, poor tissue compliance, and worsening of cardiac dysfunction. According to the location of cardiac scars and underlying cause (4, 5), cardiac fibrosis can be classified into various forms, among which reactive interstitial fibrosis and replacement fibrosis are the most relevant type of the ischemic adult heart. Being the major cell type in the adult myocardium, CFs perform a critical role in maintaining ECM protein homeostasis. The activation of CFs can lead to the transition into myofibroblasts, which is a critical step in the development of cardiac fibrosis. Besides CFs, there are various types of cells involved in the process of cardiac fibrosis *via* different pathways. It is widely known that cardiac fibrosis can provoke chamber dilation, cardiomyocyte hypertrophy, and apoptosis and finally result in congestive heart failure (6–8). Therefore, it is essential to discover potential diagnostic or therapeutic targets for cardiac fibrosis.

Exosomes, ranging in 30–150 nm of size, play an essential role in cellular communications by their bioactive contents (9, 10). As a cell-free system, exosomes could lead to improvement in cardiac function without triggering an immune response by including cardioprotective components such as miRNAs and proteins, emerging as an appropriate candidate for cardiac fibrosis treatment. Recent research studies show that inhibiting exosome secretion or targeting specific molecules in CF-derived exosomes could be a promising therapeutic strategy in ischemic heart disease (11, 12). In this review, we demonstrate the current understandings of the cellular effectors, molecular pathways, and exosomal roles in cardiac fibrosis.

## Cellular Effectors of Cardiac Fibrosis

**Figure 1:**
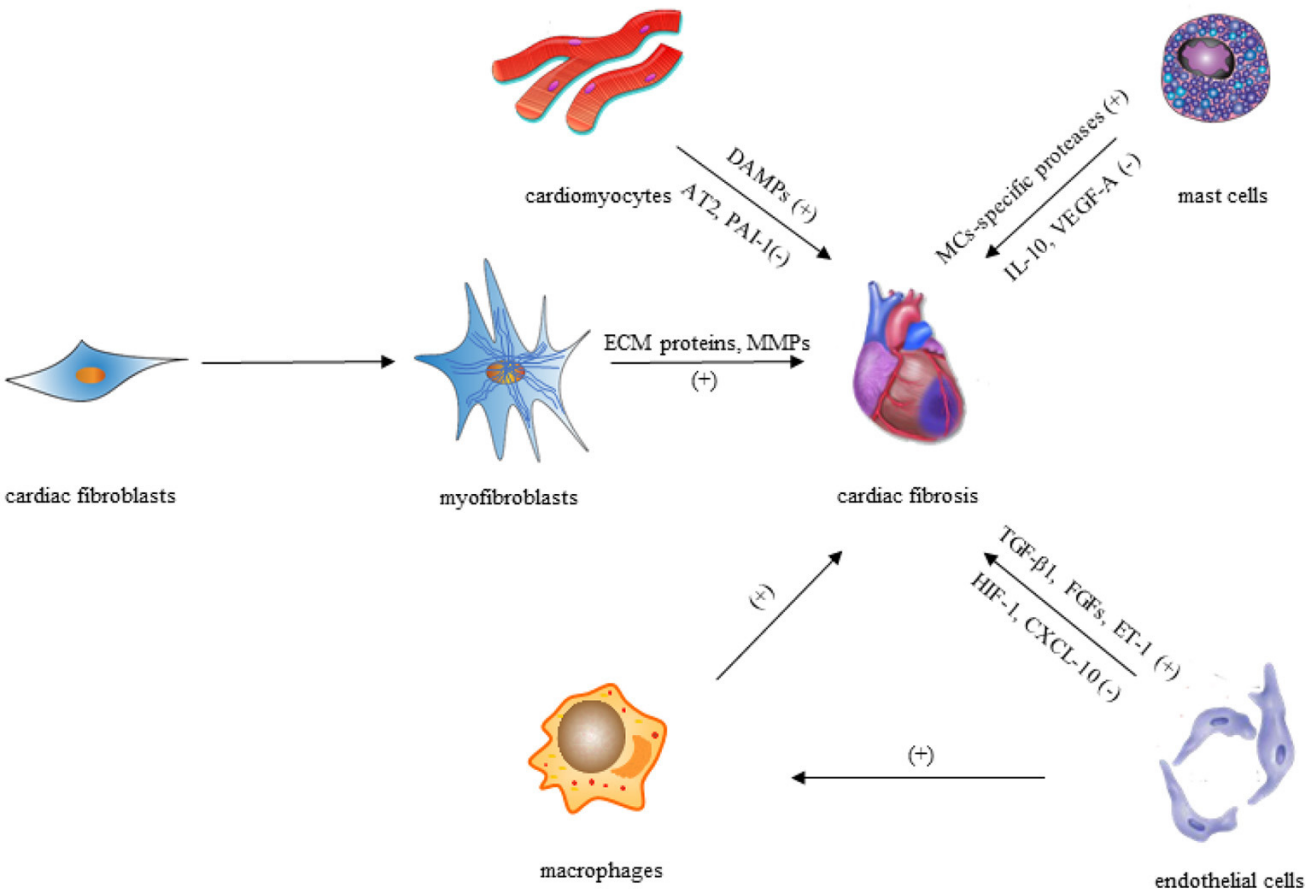
Cellular effectors of cardiac fibrosis.

After a myocardial injury, CFs convert to their activated form (termed as myofibroblasts) by upregulating expression of pro-inflammatory cytokines, which is defined as the key cellular event in cardiac fibrosis. Though activated myofibroblasts have been the primary effector cells in the fibrotic heart by producing ECM proteins directly, macrophages/monocytes, mast cells (MCs), lymphocytes, cardiomyocytes, and vascular cells (Figure 1) can also play vital roles in the fibrotic response *via* secretion of a variety of fibrogenic mediators such as matricellular proteins and growth factors.

### Fibroblasts and Myofibroblasts

**Figure 2:**
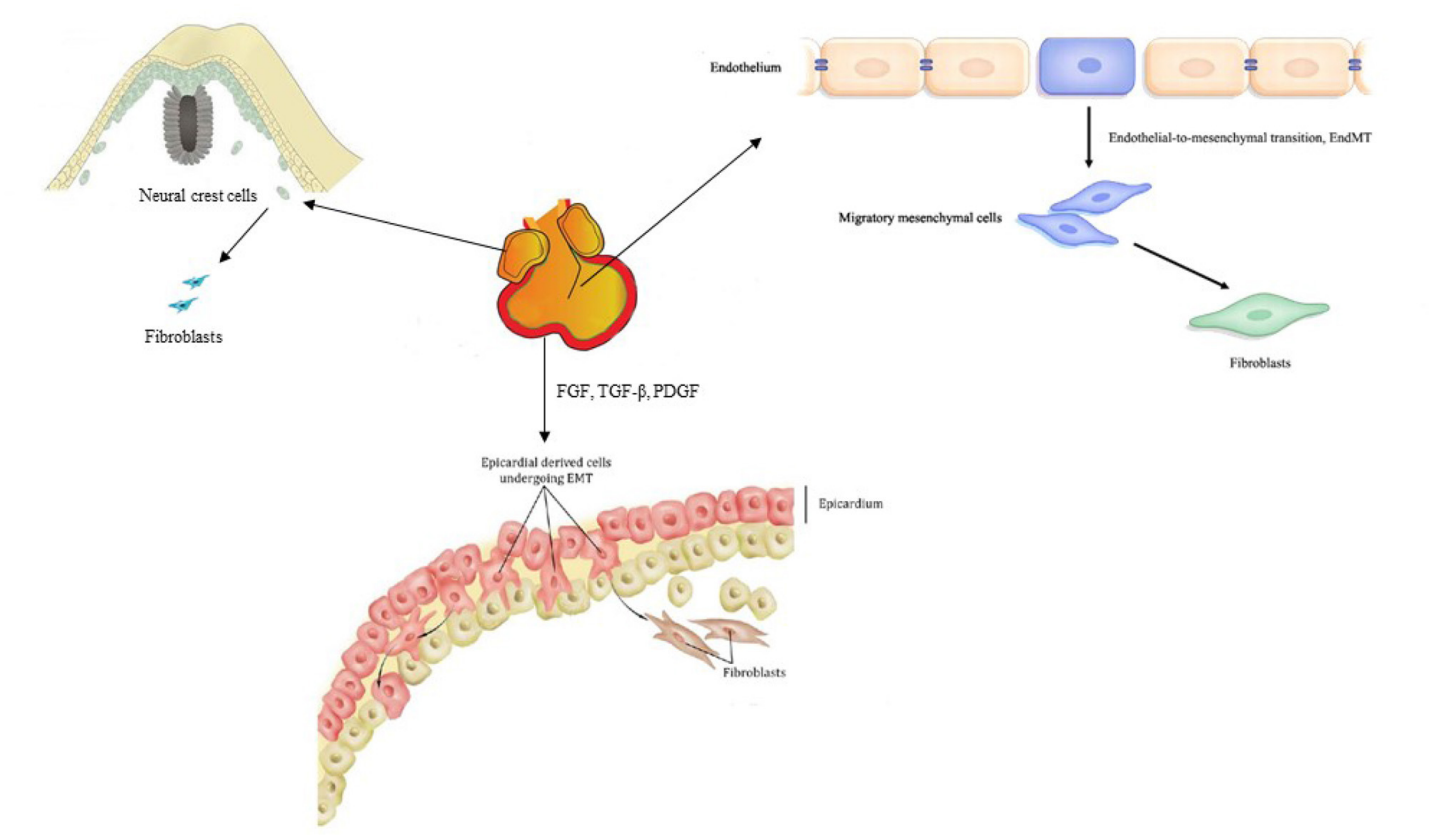
Origin of cardiac fibroblasts.

It is recognized that the transdifferentiation from CFs to myofibroblasts is the core cellular event in cardiac fibrosis. In order to clarify the role of CFs and myofibroblasts in cardiac fibrosis, several markers have been found to identify and distinguish CFs and myofibroblasts (13–37) (Table 1). It has been clear that most CFs derived from the epicardium, a protective epithelial layer that entirely covers the four cardiac chambers, undergoing epithelial–mesenchymal transition (EMT) (38, 39). Smaller populations are derived from the endocardium (40, 41) and cardiac neural crest (20) and are mostly found in the interventricular septum and right atrium, respectively (Figure 2). However, the origin of the myofibroblasts forming fibrotic lesions in failing hearts has been a source of debate. Most investigations in the last 10 years have revealed that activated myofibroblasts in remodeling and the infarcted hearts are primarily derived from resident CFs (20), and it is well-established that the transformation of CFs to myofibroblasts is a core cellular event involved in fibrotic response under cardiac injury. Cardiac myofibroblasts, a contractile and secretory cell type, not only contribute to the structure of ECM proteins in fibrotic hearts but also play an important role in matrix remodeling regulation through the production of proteases including the matrix metalloproteinases (MMPs) as well as their inhibitors.

**Table 1:** Summary of molecular markers used for the identification of cardiac fibroblasts and myofibroblasts.

**Biomarker**	**Location**	**Function**	**Expression in cardiac fibroblast**	**Expression in cardiac myofibroblast**	**Expression in other cell types**	**References**
Discoidin domain receptor 2 (DDR2)	Cell surface	Collagen-specific receptor tyrosine kinase mediating cell growth, migration, and differentiation	Yes	Yes	Epicardium	(13–18)
Vimentin	Cytoskeletal	Intermediate filaments for motility and cell shape	Yes	Yes	Endothelial cells, macrophages	(19–22)
Fibroblast-specific protein 1 (FSP1)/S100 calcium-binding protein A4 (S100a4)	Cytosolic	Calcium-binding protein for motility and tubulin polymerization	Yes	Unknown	Immune cells	(23, 24)
Thymus cell antigen 1 (Thy1, CD90)	Cell surface	Membrane glycoprotein for cell adhesion	Yes	No	Immune cells, lymphatic endothelial cells and pericytes	(25–27)
The transcription factor 21 (TCF21)	Nucleus	Regulates mesenchymal cell transitions	Yes	Yes	Epicardium	(25, 28, 29)
Platelet-derived growth factor receptor α (PDGFR α)	Cell surface	Tyrosine kinase receptor	Yes	Unknown	Platelets, epicardium	(30)
Collagen 1α1-GFP	Transgene	Targeting collagen I protein-producing cells	Yes	Unknown	Endothelial and vascular smooth muscle cells	(22, 32)
α-Smooth muscle actin (α-SMA)	Cytoskeletal	Intermediate filament-associated protein for cell contraction	No	Yes	Epicardium, smooth muscle cells, pericytes, and cardiomyocytes	(33, 34)
Periostin	Extracellular matrix (ECM)	Cardiac development, remodeling and ECM organization	No	Yes	Epicardium, vascular smooth muscle cells, and valve interstitial cells	(35–37)

**Figure 3:**
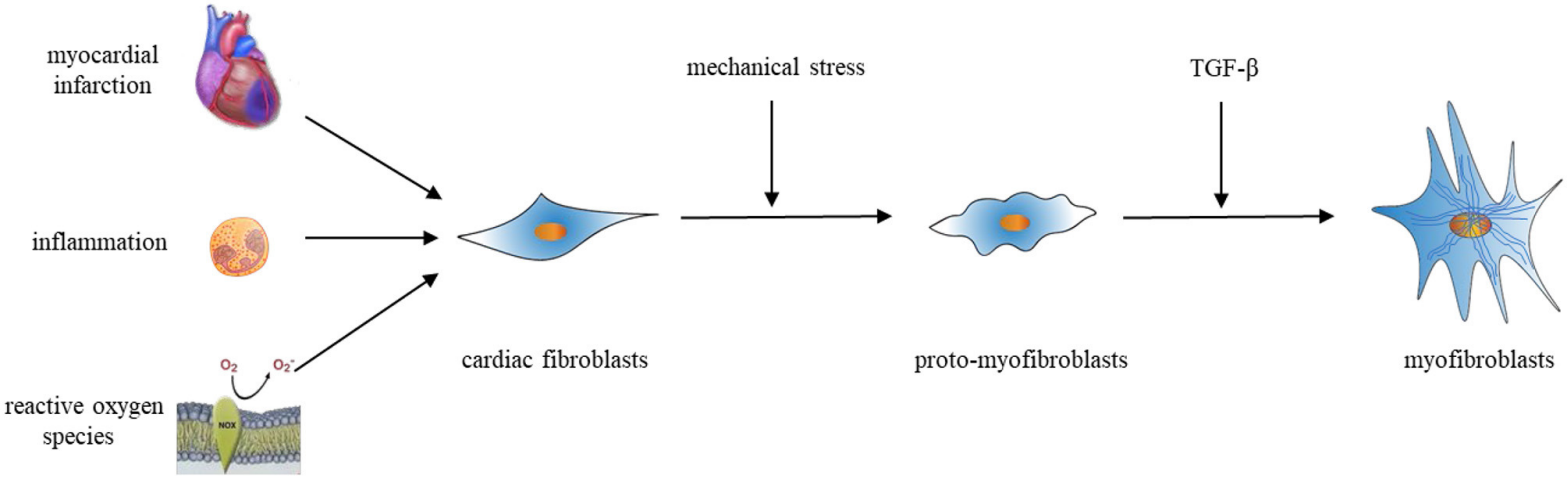
Activation of cardiac fibroblasts.

So far, few factors that can independently induce CF activation have been identified. Evidence has revealed that, following mechanical stress, fibroblasts change to proto-myofibroblasts (an intermediate cell) (42). Proto-myofibroblasts contain unique cell markers including a splice variant of fibronectin called the fibronectin extradomain A (ED-A) and stress fibers (43). The cytokine TGF-β further stimulates proto-myofibroblasts, causing them to develop into the myofibroblast cell phenotype; thus, it, in turn, leads to heart failure associated with cardiac remodeling. Inflammation, MI, changes in mechanical tension, reactive oxygen species (ROS), age, and other factors can all alter the activation of CFs (Figure 3). We will discuss the specific molecular pathways contributing to CF activation in the remodeling heart in section Fibrogenic growth factors.

### The Monocytes/Macrophages

According to increasing evidence, macrophages and monocytes have both been confirmed to play vital roles in the regulation of cardiac fibrosis. Macrophages and monocytes in the injured heart appear to be increasingly heterogeneous depending on their different subpopulation (44), and their phenotypic and functional flexibility allow them to perform diverse functions in fibrotic responses, such as serving as a primary source of fibrogenic growth factors and cytokines, producing matricellular proteins, and secreting matrix remodeling proteases (45). Moreover, circulating fibroblast progenitors may be implicated in the progression of cardiac fibrosis as suggested by numerous studies using bone marrow transplantation techniques (46). These hematopoietic progenitors could be monocyte subsets that can differentiate fibroblasts, comparable with the CD14+ “fibrocytes” discovered in humans (47), which imply that macrophages and monocytes in fibrotic hearts could be sources of myofibroblasts.

### The Mast Cells

MCs are innate immune cells found almost everywhere of the body, including the heart. Resident MCs in the heart can respond to damage-associated molecular patterns (DAMPs) after injury, thus influencing the development of cardiac remodeling. However, the precise function of MCs in cardiac fibrosis is debatable, as the secretory proteins produced by MCs can be both anti- and profibrotic in nature. MC-specific proteases such as chymase and tryptase released by degranulation could induce TGF-β1 production (48–50), which plays a role in cardiac fibrosis through collagen synthesis, myofibroblast differentiation, and fibroblast stimulation (1, 51). In addition, cytokines like tumor necrosis factor (TNF) (52) and interleukin (IL)-1β (53) stored in MC granule (MCG) can also promote cardiac fibrosis through cardiomyocyte apoptosis during degranulation (54).

MCs, on the other hand, secrete anti-inflammatory mediators including IL-10 (55), which has been shown to inhibit excessive cardiac remodeling by activating STAT3 and suppressing NF-κB (56–58). Besides, MCs can produce vascular endothelial growth factor (VEGF)-A (52, 53), as one of the important anti-fibrotic mediators, which can increase capillary density in damaged tissues and promote proper repair in cardiac fibrosis (59–61). Over the past few years, various studies have been carried out for investigating the functions of MCG in fibrosis. MCG therapy of mesenchymal stem cells (MSCs) *in vitro* reduced TGF-β1-mediated transition of MSCs to myofibroblasts, while *in vivo* delivery of MCGs from rats to the myocardium during AMI lowered fibrosis and enhanced capillary density (62). These findings suggest that MCs have anti-fibrotic properties and could be used as therapeutic targets in cardiac remodeling.

### The Endothelial Cells

The prevalence of perivascular fibrosis in the injured heart may indicate that endothelial cells are involved in cardiac fibrosis (63). Under pathophysiologic conditions, endothelial cells may enhance fibrotic responses *via* a variety of mechanisms after the myocardial injury. First, several profibrotic mediators produced by endothelial cells, such as FGFs, TGF-1, and endothelin (ET)-1, may play key roles in the development of cardiac fibrosis (64, 65). Second, endothelial cells may produce pro-inflammatory cytokines and chemokines, contributing to recruitment of lymphocytes and macrophages with fibrogenic actions (66). Third, although low numbers of endothelial-derived fibroblasts were detected in the remodeling myocardium, endothelial cells may undergo endothelial to mesenchymal transition (EndMT), increasing the number of fibroblasts (34).

On the contrary, anti-fibrotic mediators could also be produced by endothelial cells. Endothelial cells have been found to express hypoxia-inducible factor (HIF)-1 for protecting the pressure-overloaded myocardium from fibrosis *via* suppression of TGF-β signaling partially (67). Furthermore, endothelial cells exert inhibitory actions on cardiac fibrosis by producing and secreting interferon-γ-inducible protein (IP)-10/CXCL10, a CXC chemokine that prevents the migration of CFs in the infarcted heart (68).

### The Cardiomyocytes

The roles of cardiomyocytes in the process of cardiac fibrosis are two sides of the coin. For one thing, cardiomyocytes may promote interstitial fibrosis through neurohumoral and growth factor-mediated pathways, such as cardiomyocyte-specific mineralocorticoid receptor signaling (69), TGF-β receptor II (TβRII) signaling (70), and insulin-like growth factor (IGF)-1 signaling (71). Moreover, necrotic cardiomyocytes trigger an inflammatory response that finally leads to activation of fibroblasts *via* release of DAMPs, which means cardiac fibrosis may occur due to cardiomyocyte death, instead of cardiomyocyte-derived fibrogenic signals (51). For another, cardiomyocyte-specific overexpression of angiotensin II (Ang II) type 2 (AT2) receptor or the plasminogen activator inhibitor (PAI)-1 exerts anti-fibrotic actions *via* the kinin/NO system activation or inhibition of TGF-β synthesis, respectively (72, 73).

## Molecular Pathways in Cardiac Fibrosis

The complexity of interconnections and the extensive range of molecular pathways involved in the fibrotic response have restricted our understanding of the mechanism of cardiac fibrosis. High-throughput transcriptomic and genomic techniques have recently been employed to find new molecular signals and pathways linked to the fibrotic response's initiation, regression, and progression (74); and in the development of cardiac fibrosis, various molecular routes have been identified. Most fibrotic heart diseases, regardless of cause, appear to include the aldosterone/angiotensin axis and fibrogenic growth factors such as platelet-derived growth factor (PDGF) and TGF-β. Moreover, several inflammatory signals (3, 75) such as TNF-α and IL-6 may regulate reparative and ischemic fibrosis by transducing the cascades of intracellular signaling that result in the transcription of ECM genes and translation of matrix remodeling-related proteins. Here, we demonstrate signaling pathways and mediators known to influence process of cardiac fibrosis after myocardial injury, hoping to find novel therapeutic targets or strategies.

### Neurohumoral Pathways

#### The Renin–Angiotensin–Aldosterone System

During the progression of cardiac fibrosis, the renin–angiotensin–aldosterone system (RAAS), of which Ang II appears to be the primary effector, is persistently engaged. In fibrotic hearts, the oligopeptide Ang II, which induces vasoconstriction and high blood pressure, is raised. Angiotensin-converting enzyme (ACE) and renin, which are required for the production of Ang II, are produced by fibroblasts and macrophages invading the damaged heart (76, 77). Both *in vivo* and *in vitro* investigations suggest that Ang II is involved in TGF signaling. TGF-1 expression is induced by Ang II in fibroblasts and cardiomyocytes *via* the Ang II type 1 (AT 1) receptor, which plays a crucial role in profibrotic signaling (78–80); and *in vivo*, TGF-β is necessary for Ang II to induce both cardiac fibrosis and hypertrophy (81, 82). Besides, Ang II is also intimately involved with the inflammatory response, and in CFs, Ang II enhances their collagen-synthetic activity through extracellular signal-regulated kinase by an IL-6-dependent mechanism indeed. Another mechanism underlying the fibrotic capability of Ang II could involve miR-29b. *In vitro*, miR-29b suppression promotes Ang II-induced collagen type I and α-SMA expression, but overexpression of miR-29b inhibits it. It is indeed possible that miR-29b targets a sequence within the TGF-β1 coding area, which explains this observation. On the contrary, AT2 signaling may inhibit AT1-mediated actions, suppressing CF proliferation and matrix synthesis, serving as a negative regulator of Ang II-mediated profibrotic responses.

Aldosterone is also capable of inducing fibrotic responses in the myocardium after cardiac injury, suggested by patients with adrenal adenomas and experimental animal studies. Several potential mechanisms have been involved in the profibrotic activities of aldosterone in the heart. First, aldosterone may have pro-inflammatory effects on vascular cells by increasing the production of cytokines like TNF-α *via* NF-κB activation. Second, aldosterone may induce a fibrogenic phenotype in macrophages *via* the mineralocorticoid receptor. Third, aldosterone may activate cardiomyocyte-derived fibrotic signals, involving regulation of MMP-2/9 activity and the TGF-β-connective tissue growth factor profibrotic pathway. Fourth, aldosterone may exert a direct effect on CFs, stimulating proliferation and increasing collagen synthesis.

#### GPCR/Adrenergic Signaling

It has been reported that activation of adrenergic signaling *via* β-adrenergic receptor (AR) can induce cardiomyocyte death and subsequent reparative fibrosis, thus leading to cardiac remodeling. Although there are several subtypes of β-AR expressed in the heart, the predominant form of β2-AR seems to be expressed on CFs. Collagen secretion, cell proliferation, migration, and transformation to the myofibroblast phenotype can all be induced by direct activation of β2-AR on CFs, mediated through p38 MAPK signaling partially. In addition, β-AR signaling can also regulate cytokine expression by macrophages and induce growth factor synthesis by cardiomyocytes, which plays an important role in promoting cardiac fibrosis. However, not all types of β-ARs are involved in the profibrotic responses. On the contrary, several studies have proved that, in a model of pressure overload-induced cardiac fibrosis, β3-AR signaling in cardiomyocytes may protect the heart, due to downregulation of the matricellular protein CCN2 by cardiomyocytes.

Adrenergic stimulation causes structural changes in G protein βγ subunits in the damaged myocardium, culminating in activation of G protein-coupled receptor kinase 2 (GRK2). In an experimental model of MI, GRK2 activation in CFs has been shown to have substantial fibrotic effects. Although the specific fibrotic signals activated by GRK2 remain unclear, GRK2 represents a critical target for therapeutic interventions against cardiac fibrosis.

#### Endothelin-1

The endothelin family of peptides was mostly known for its vasoconstriction capabilities; however, it is now being recognized for its potential role in tissue fibrosis. ET-1, one of the significant endothelin isoforms in humans, is thought to be secreted predominantly by endothelial cells but also can be produced by other cells including fibroblasts, cardiomyocytes, and macrophages. The ETA and ETB receptors, which have been found to perform opposite roles, are two recognized ET-1 receptors in the heart. At first, it was thought that these two receptors were only expressed on endothelial cells; however, the latest evidence suggests (83, 84) that they can also be expressed in other types of cells such as macrophages, cardiomyocytes, and CFs.

Both *in vitro* and *in vivo* studies suggest that ET-1 appears to be a potent fibrogenic mediator. *In vitro*, ET-1 enhances proliferation and collagen production in isolated human CFs *via* ETA receptor; *in vivo*, overexpression of ET-1 in the heart induces myocardial fibrosis associated with biventricular systolic and diastolic dysfunction. In addition to fibroblast-activating characteristics of its own, ET-1 can also act as a downstream of cytokines and neurohumoral mediators such as TGF-β and Ang II, serving as a link between fibrosis and inflammation. For example, the development of cardiac fibrosis in response to Ang II is impaired in mice with vascular endothelial cell-specific ET-1 deficiency, regulated by the myocardin-related transcription factor (MRTF)-A.

Moreover, endothelin antagonists are now approved to treat pulmonary hypertension, and many believe they will also be beneficial in the treatment of heart pathological fibrosis. Bosentan, a non-selective endothelin receptor antagonist routinely used to treat pulmonary hypertension, has also been shown to reduce fibrotic myocardium remodeling in hypertensive and reparative cardiac fibrosis animal models (85). Despite the failure of several randomized controlled studies exploring the impact of endothelin antagonists in heart failure and coronary artery disease, manipulating ET-1 signaling appears to be promising. More researches are needed to explore whether ET-1 and its receptors may be appropriate clinically viable anti-fibrotic treatment targets.

### Fibrogenic Growth Factors

#### TGF-β

The TGF-β family is a group of pleiotropic and multifunctional peptides activated in experimental models of cardiac fibrosis and fibrotic human hearts markedly (1, 3). TGF-β is found in three isoforms (TGF-β1, TGF-β2, and TGF-β3) in mammals (86), among which TGF-β1 acts as the predominant isoform in the cardiovascular system and expresses ubiquitously. In the injured heart, TGF-β1, which is present in the normal heart as a latent complex, is transformed from the latent form to the active form *via* a variety of mediators. Proteases, including MMP-2, MMP-9, and plasmin, are widely acknowledged to participate in the activation of TGF-β as well as the matricellular protein thrombospondin 1 (TSP-1) (1), which plays an important role in cardiac remodeling. Upon activation, a group of studies have revealed that TGF-β was involved in the pathogenesis of cardiac fibrosis through Smad-mediated pathways where TGF-β binds to the constitutively active TβRII on the cell surface, transphosphorylates the cytoplasmic domain of the type I receptor (TβRI), and then gets connection with the Smads; or through Smad-independent pathways, in which TGF-β/TAK-1 signaling may exert profibrotic actions (1). Meanwhile, negative regulation of TGF-β signaling may be crucial in preventing cardiac fibrosis. A study conducted in a mouse model of pressure overload-induced heart failure has suggested that cleavage and release of a soluble endoglin may inhibit fibrogenic actions of TGF-β (87).

In addition, TGF-β is a critical fibrogenic mediator that may have the potential to affect all cell types involved in cardiac fibrotic response. MCGs are known to contain TGF-β in a large amount, while TGF-β-induced Smad-dependent pathways are activated by MC chymase, which results in fibrogenic effects (88). Besides, profibrotic growth factors including TGF-β can be produced and secreted in significant quantities by macrophages and monocytes. In return, TGF-β-mediated actions of these cell types may also play a paracrine role in fibrotic response. Moreover, endothelial cells may promote fibrotic cardiac remodeling through the expression of profibrotic mediators, such as TGF-β1, FGFs, or ET-1.

What is more, TGF-β-stimulated myofibroblast transdifferentiation is induced by activation of the Smad3 signaling cascade, which promotes α-SMA transcription in fibroblasts (89) and enhances ECM protein synthesis. Furthermore, cardiomyocyte-specific TβRII knockdown significantly reduced fibrosis in the pressure-overloaded heart (70), implying that cardiomyocyte-specific TGF-β signaling is essential in the pathogenesis of fibrotic remodeling.

#### Platelet-Derived Growth Factor

The PDGF family includes homo- or hetero-dimeric growth factors (such as PDGF-AA, PDGF-BB, PDGF-AB, PDGF-CC, and PDGF-DD) that signal *via* two distinct receptors: PDGFR-α and PDGFR-β (1). *In vivo*, PDGF-A and PDGF-C bind to PDGFRα, while PDGF-B and PDGF-D bind to PDGFRβ in general (90). PDGF-B and PDGF-D are expressed by endothelial cells, whereas PDGFRβ is expressed by vascular mural cells (pericytes and smooth muscle cells). Myocardial cells express both PDGF-A and PDGF-C, while PDGFRα-positive interstitial cells have been found in the myocardium, epicardium, and endocardium (90). With pleiotropic effects of PDGF signaling, all PDGFs have been reported to play a certain role in the development of cardiac fibrosis. Overexpression of PDGF-C (91) and PDGF-D (92) from the α-myosin heavy chain promoter (α-MHC), as well as PDGF-A (both splice variants) and PDGF-B, has been reported to generate cardiac fibrosis and hypertrophy in transgenic mice, though the degree and location of fibrosis vary between the different ligands (90). Besides, a group of studies suggested that PDGF stimulates fibroblast proliferation and differentiation to myofibroblasts *in vitro*, whereas PDGF blockade reduces interstitial fibrosis of the infarcted hearts in rats and suppresses atrial-selective canine fibroblast activation, removing the distinctive atrial–ventricular fibroblast activation differences (93). Moreover, a study implied that PDGF may also act to promote fibrosis by elevating TGF levels, for it can significantly upregulate profibrotic TGF-1 mRNA and accelerate cardiac fibrosis and arteriosclerosis when three of the isoforms, PDGF-A, PDGF-C, or PDGF-D, was introduced into the heart using adenovirus-mediated delivery (94). Similarly, PDGFRα appears to be a strong CF marker, possibly implicated in the production of CFs from epicardium, while PDGFR-β regulates the development of vascular smooth muscle cells from epicardium-derived cells (22, 24). Injection of a neutralizing PDGF receptor-specific antibody was also shown to reduce atrial fibrosis in several studies (95). These findings strongly imply that PDGF and PDGFR could be useful targets for anti-fibrotic treatment in the heart.

### Inflammatory Cytokines

#### Tumor Necrosis Factor-α

TNF-α is a powerful pro-inflammatory cytokine that exerts pleiotropic effects on a variety of cell types and is reported to be crucial in the process of cardiac fibrosis. Transmembrane TNF-α, a precursor of the soluble TNF-α, is expressed on activated lymphocytes and macrophages as well as other cell types, exerting its biological actions by binding to type 1 and 2 TNF receptors (TNF-R1 and TNF-R2) (96), which can play different roles. Studies have shown that TNF-α in deficient mice after non-reperfused MI exacerbates cardiac remodeling, hypertrophy, NF-κB activity, and inflammation as well as border zone fibrosis through TNF-R1, whereas it ameliorates these events through TNF-R2 (97). In addition, an increasing number of studies suggest that cardiac fibrosis is promoted by TNF-α *via* a range of mediators and the interaction with other cell types. Heart failure was accelerated in transgenic mice by cardiac-specific overexpression of TNF-α, which was associated with increased collagen synthesis, deposition, and denaturation, and dramatically elevated MMP-2 and MMP-9 activities (98). Studies have also shown that fibrotic remodeling in the TNF-α overexpressing heart is associated with increased expression of TGF-βs and the interactions between CFs and MCs (98). Complementally, in models of heart pressure overload induced by Ang II infusion or aortic banding, it is demonstrated that global genetic deletion of TNF-α reduced interstitial and perivascular fibrosis (99).

#### Interleukin-6

**Figure 4:**
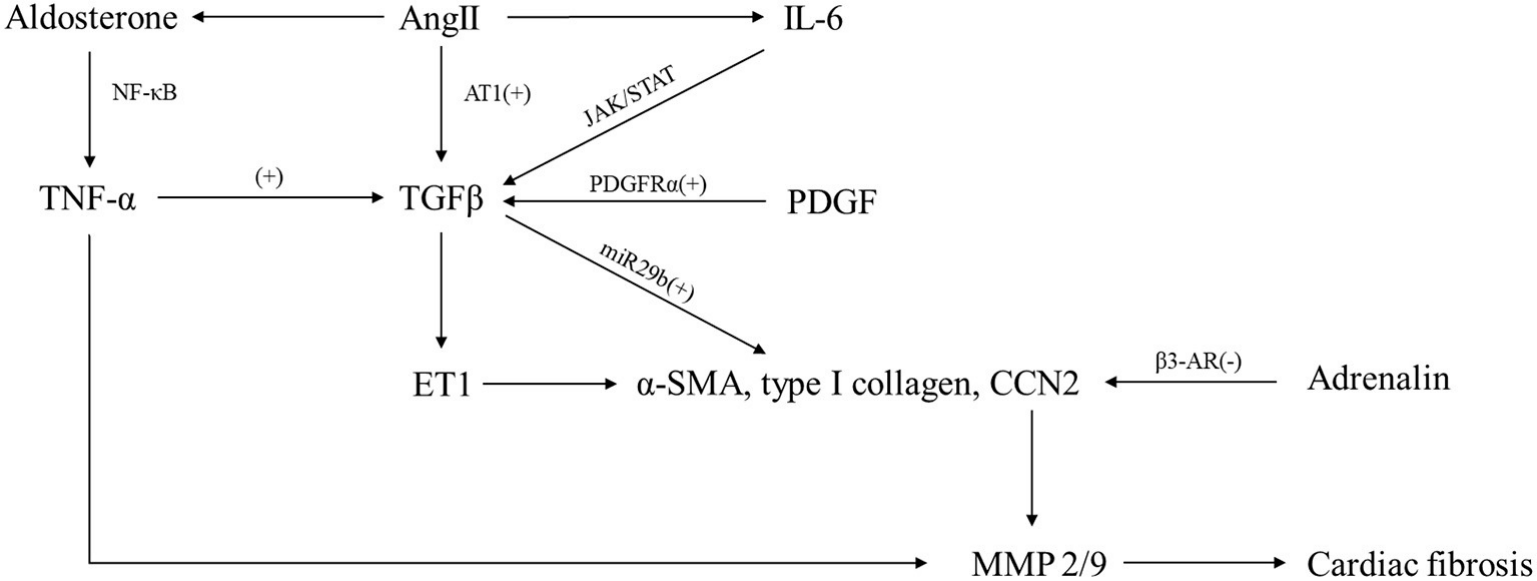
Molecular pathways participated in cardiac fibrosis.

IL-6 is a pleiotropic cytokine that has a wide range of biological functions in hematopoiesis, immunological regulation, inflammation, and cardiac fibrosis. It was first identified as a B-cell differentiation factor (100). Secreted by various types of cells, IL-6 influences a group of cell types and exerts its multiple biological activities through two different signaling pathways: classic signaling and trans-signaling. Both intracellular signaling pathways involve the signal transducer and activator of transcription (STAT) pathway and Janus kinase (JAK) pathway, though they are activated following interaction of signal transducing membrane-bound IL-6R (mIL-6R), soluble IL-6R (sIL-6R), or glycoprotein (gp130) (100, 101). Emerging evidence suggests that IL-6, as a multifunctional cytokine, has a role in cardiac fibrosis. A study using the animal model suggested that elevated production of IL-6 induced by aldosterone could further promote collagen production and cardiac hypertrophy *via* the IL-6 trans-signaling pathway (102). Similarly, increased IL-6 levels and ROS generation in rats could activate the renin–angiotensin system (RAS) and JAK1/2-STAT1/3 signaling pathways, thus ultimately leading to activation of TGF-1β/Smad3 fibrotic pathway (103). Moreover, a study of neonatal rats under hypoxic conditions showed that overexpression of IL-6 was sufficient for inducing myofibroblastic proliferation, differentiation, and fibrosis, probably through improved TGF-β1-mediated MMP-2/MMP-3 signaling (102). Furthermore, IL-6 is a downstream signal of hypoxia-induced mitogenic factor (HIMF), and it plays a key role in cardiomyocyte hypertrophy and cardiac fibrosis *via* the MAPK and CaMKII-STAT3 pathways (104). Directly, by activating CFs to secrete Tenascin-C (TN-C), ET-1, and collagen, IL-6 produced by macrophages can also cause cardiac fibrosis (105). However, different studies on the role of IL-6 in cardiac fibrosis can be conflicting. In models of left ventricular pressure overload, genetic loss of IL-6 reduced cardiac dysfunction and fibrosis, whereas another study utilizing a model of pressure overload caused by transverse aortic constriction found no effect of germline IL-6 loss on ECM protein deposition and cardiac fibrosis (98). Therefore, we conclude that IL-6 and IL-6Rs may act as therapeutic targets of cardiovascular disease in the near future (Figure 4).

## Role of Exosomes in Cardiac Fibrosis

Exosome-mediated intercellular signaling, which can transfer various functional modulators including proteins, lipids, and RNA, plays an increasingly important role in cardiovascular diseases. CFs are major components of the heart, ischemia/hypertrophy activates these fibroblasts, and they are involved in cardiac fibrosis and remodeling (106). Post-cardiac injury, fibroblast-derived miR-21-enriched exosomes can lead to cardiac myocyte hypertrophy and remodeling (11). In addition, miR-155 enriched in macrophage-derived exosomes led to enhanced proliferation and differentiation of resident fibroblasts and further exacerbated inflammation (12). Furthermore, exosomes *via* use of a targeting cardiac homing peptide or encapsulated in functional peptide hydrogels exhibit better ability in improving cardiac function and reducing fibrosis (107, 108). Besides, changes of miRNAs or proteins in exosomes derived from plasma or peripheral blood are considered as novel biomarkers for cardiac fibrosis or cardiac remodeling. Therefore, exosomes could be potential therapeutic treatments in cardiac fibrosis. Thorough knowledge of exosomes and exosome-mediated intercellular communication in cardiac fibrosis will provide better understanding to develop novel strategies for cardiac fibrosis treatments.

### Exosomes: Biogenesis, Isolation, and Uptake

In response to different physiological states, exosomes are secreted by various cell types, such as MCs (109), macrophages, CFs, and exogenous MSCs, whose size range from 30 to 150 nm. Initially, transmembrane proteins are endocytosed and trafficked to early endosomes (EEs). EEs then mature into late endosomes (LEs) and generate intraluminal vesicles (ILVs) in the lumen of the organelles. Multivesicular bodies (MVBs), namely, LEs containing these ILVs, can fuse with plasma membrane and release exosomes into the extracellular space or fuse with lysosomes and degrade exosomes (110, 111). Different cell type- and microenvironment-derived exosomes transport distinct proteins, lipids, and nucleic acid cargoes (112, 113). Generally, exosomes are formed with tetraspanin family (CD9, CD63, and CD81) transmembrane proteins, tumor susceptibility gene 101 (TSG101), major histocompatibility complex (MHC) class II molecules, programmed cell death 6-interacting proteins (PDCD6IPs), heat shock proteins (HSPs) (HSP60, HSP70, and HSP90), cytoskeletal proteins (actin and tubulin), annexins (regulate cytoskeletal changes in membranes and membrane fusion), and membrane transport proteins (114).

Different techniques including microfiltration, gel filtration, ultracentrifugation, and commercial exosomes isolation kits are used to isolate exosomes from body fluids, plasma, or cell culture medium (115). Among these, ultracentrifugation is regarded as the gold standard for exosomes isolation and is also the most common method. Exosomes can enter recipient cells *via* distinct mechanisms including lipid membrane fusion, internalization by receptor-mediated endocytosis, receptor-mediated binding, and activation of downstream signaling (116). Total understanding of the biogenesis, isolation, and uptake of exosomes may contribute to find novel strategies for the treatment of cardiac fibrosis.

### Exosome Contents for the Treatment of Cardiac Fibrosis

#### MicroRNAs

MiRNAs, small endogenous oligonucleotides of 21–25 nucleotides, are critical in regulating post-transcriptional gene. Additionally, exosomes, containing different numerous miRNAs, could contribute to or alleviate a variety of pathologies including cardiac fibrosis. Exosomes, derived from distinct cell types including fibroblasts and exogenous MSCs, with upregulation or downregulation of certain miRNAs, can exhibit better ability in attenuating cardiac fibrosis and improving cardiac function (Table 2).

**Table 2:** MiRNAs and proteins involved in exosomes for the treatment of cardiac fibrosis.

**Name**	**Level**	**Derivation**	**Disease**	**Target gene/pathway**	**Effects**	**References**
**MiRNAs**						
MiR-21-3p	Downregulation	Cardiac fibroblasts	Heart failure (HF)	Orbin and SH3 domain-containing protein 2 (SORBS2) PDZ and LIM domain 5 (PDLIM5)	Cardiac hypertrophy↓	(11)
MiR-27a, MiR-28-3p, MiR-34a	Upregulation	Cardiac fibroblasts	HF	Nuclear factor erythroid 2-related factor 2 (Nrf2)	Oxidative stress↑ Cardiac remodeling↑	(117)
MiR-155	Upregulation	Macrophages	Uremic cardiomyopathy	Forkhead transcription factors of the O class (FoxO3a)	Cardiomyocyte pyroptosis↑ Cardiac hypertrophy and fibrosis↑	(118)
MiR-19a-3p	Upregulation	Endothelial cells	MI	MiR-19a-3p/Thrombospondin 1	Vascularization↑ Myocardial fibrosis↓ Left ventricular ejection fraction↑	(119)
MiR-133	Upregulation	Endothelial cells	Myocardial fibrosis	Y box binding protein 1 (YBX-1)	Angiogenesis↑ mesenchymal-endothelial transition of cardiac fibroblast↑	(120)
MiR-10b-5p	Upregulation	Endothelial cells	MI	SMAD-specific E3 ubiquitin protein ligase 1 (Smurf1) Histone deacetylase 4 (HDAC4)	Cardiac fibroblast activation↓	(121)
MiR-29b, MiR-455	Upregulation	Cardiomyocytes	Diabetes	Matrix metalloproteinase 9 (MMP-9)	Fibrosis and myocyte uncoupling↓	(122)
MiR-378	Upregulation	Cardiomyocytes	Myocardial fibrosis	Mitogen-activated protein kinase kinase 6 (MKK6)/P38 MAPK pathway	Fibrosis↓	(123)
MiR-208a	Upregulation	Cardiomyocytes	Cardiac fibrosis	Dual-specificity tyrosine phosphorylation-regulated kinase 2 (Dyrk2)	Cardiac fibroblast↑ Myofibroblast differentiation↑ Cardiac fibrosis↑	(124)
MiR-19a	Upregulation	Mesenchymal stem cells (MSCs)	MI	Phosphatase and tensin homolog (PTEN)/Akt pathway	Infarct size↓ Fibrosis↓ Cardiac function↑	(125)
MiR-210	Upregulation	MSCs	MI	MiR-210/hypoxia-inducible factor-1 α (HIF-1α)	Fibrosis↓ Angiogenesis↑ Apoptosis↓	(126)
MiR-22	Upregulation	MSCs	MI	Methyl CpG binding protein 2 (Mecp2)	Cardiac fibrosis↓ Anti-apoptosis↑	(127)
MiR-24	Upregulation	Human umbilical MSCs	MI	MiR-24/Bim pathway	Cardiac fibrosis↓ Cardiac function↑	(128)
MiR-26a	Upregulation	Satellite cells	Uremic cardiomyopathy	FBXO32/atrogin-1 TRIM63/MuRF1	Cardiac fibrosis lesions↓	(129)
MiR-29c	Upregulation	Placenta-derived MSCs	Duchenne muscular dystrophy	TGF-β	Fibrosis in the diaphragm and cardiac muscles↓ Inflammation↓ Utrophin↑	(130)
MiR-92a	Upregulation	Cardiosphere-derived cells (CDCs)	MI	Bone morphogenetic protein 2 (BMP2)	Contractility↑ Fibrosis↓	(131)
MiR-126	Upregulation	Adipose-derived stem cells (ADSCs)	MI	–	Cardiac fibrosis↓ Inflammation↓ Apoptosis↓ Angiogenesis↑	(132)
MiR-133a	Upregulation	Cardiac progenitor cells (CPCs)	MI	Bim Bmf bFgf Vegf	Apoptosis↓ Fibrosis↓ Hypertrophy↓	(133)
MiR-146a-5p	Upregulation	CPCs	Doxorubicin/ trastuzumab-induced cardiac toxicity	Traf6 Smad4 Irak1 Nox4 Mpo	Myocardial fibrosis↓ CD68+ inflammatory cell infiltrates↓ Inducible nitric oxide synthase expression↓ Left ventricular dysfunction↓	(134)
MiR-146a	Upregulation	ADSCs	MI	Early growth response factor 1 (EGR1)/TLR4/NFkB	Apoptosis↓ Inflammatory response↓ Fibrosis↓	(135)
MiR-425, MiR-744	Downregulation	Plasma	HF	TGF-β1	Collagen formation↑ Fibrogenesis↑	(136)
MiR-21	Upregulation	Human peripheral blood	MI	Smad7 PTEN MMP-2	Fibrosis↑	(137)
MiR-142-3p	Upregulation	CD4+ T cells	MI	WNT pathway	Cardiac fibrosis↑ Dysfunction↑	(138)
**Proteins**						
HSP20	Upregulation	Cardiomyocytes	Diabetic cardiomyopathy	Phosphorylated Akt Survivin SOD1	Cell death↓ Cardiac adverse remodeling↓	(139)
HSP90	Downregulation	Cardiomyocytes	Cardiac hypertrophy	STAT3	Collagen synthesis↓	(140)
Decorin Periostin	Upregulation Downregulation	Cardiomyocytes	Cardiac fibrosis	Ang II	Transformation into myofibroblast↓ Fibroblast migration↓	(141)
HSP70	Upregulation	Serum	Aging-related cardiac fibrosis	–	Fibroblast proliferation↓ Myofibroblast differentiation↓	(142)
Lamp2b Ischemic myocardium-targeting peptide CSTSMLKAC (IMTP)	Upregulation	MSCs	MI	–	Inflammation↓ Apoptosis↓ Fibrosis↓ Vasculogenesis↑ Cardiac function↑	(143)
Human antigen R (HuR)	Upregulation	Macrophages	Cardiac fibrosis	Ang II	Inflammatory and profibrogenic responses↑ Cardiac fibrosis↑	(144)
WNT3a WNT5a	Overexpression	Cardiac fibroblasts	Cardiac fibrosis	WNT pathways	Cardiac fibroblast activated↑ Cardiac fibrosis↑	(145)

*↑ means the corresponding MiRNA or protein has a positive effect on the process, or could increase the number/area of the subject; ↓ means the corresponding MiRNA or protein has a negative effect on the process, or could decrease the number/area of the subject*.

It has been confirmed that miR-21 played an essential role in fibroblast biology and that the levels were selectively increased in the failing heart, which makes it a target in heart failure (146). Bang et al. (11) revealed that miR-21 was enriched in fibroblast-derived exosomes, and the transfer of miR-21 to cardiomyocytes led to cellular hypertrophy. Additionally, Kang et al. demonstrated that miR-21-loaded human peripheral blood derived-exosomes enhanced fibrosis, making it a novel therapeutic target for cardiac fibrosis (137). Another research indicated that miR-27a-, miR-28a-, miR-34a-enriched fibroblast-derived exosomes could regulate cardiomyocyte antioxidant enzymes, thus contributing to cardiac hypertrophy (117). Therefore, exosomes derived from fibroblasts, especially those changing miRNAs contents, are a promising target for cardiac fibrosis.

Furthermore, exosomes derived from cardiomyocytes also exert therapeutic effects in cardiac fibrosis. Exosomes that contain high levels of miR-29b and miR-455 can downregulate MMP-9, thus reducing matrix degradation and mitigating fibrosis and myocyte uncoupling (122). MiR-378 secreted by cardiomyocytes mediated cardiac fibrosis *via* targeting the p38 MAPK-Smad2/3 signaling pathway and then regulating collagen and MMP expression in CFs (123). However, cardiomyocyte-derived miR-217- and miR-208-containing exosomes resulted in cardiac dysfunction and worsened cardiac fibrosis *via* targeting phosphatase and tensin homolog (PTEN) and dual-specificity tyrosine phosphorylation-regulated kinase 2 (Dyrk2) separately (124, 147). Evidence indicated that miR-142-3p-enriched exosomes derived from activated CD4^+^ T cells contributed to the activation of WNT signaling pathway and CF activation, making it a promising target for treating cardiac fibrosis post-MI (138).

Cell therapy, including different types of stem cells, has been widely considered as a therapeutic approach for the treatment of cardiac fibrosis. Placenta-derived MSCs decreased the expression of TGF-β and reduced fibrosis in cardiac muscles *via* transferring exosomal miR-29c (130). MiR-92a from CDC-derived exosomes can be enriched *via* the activation of β-catenin and contribute to attenuation of cardiac fibrosis and improved cardiac function (131).

#### Proteins

Functional proteins, as the vital contents of exosomes, also exhibit an ability in regulating cardiac remodeling and cardiac fibrosis. It is generally considered that heat shock response is a cellular intrinsic defense mechanism (148) and that the increased expression of HSPs is beneficial for cells or tissues to fight against stress stimuli and pathological conditions (149). The overexpression of HSP20 in cardiomyocytes contributes to the secretion of exosomes *via* interaction with TSG101 and leads to the elevation of HSP20 in exosomes, which remarkably improved cardiac function and attenuated adverse remodeling (139). However, myocyte-derived HSP90 exerted a profibrotic role through orchestrating the synthesis of IL-6 and activating STAT-3 in fibroblasts, leading to excess collagen secretion and deposition, thus exaggerating cardiac hypertrophy and fibrosis (140). Emerging evidence indicated that proteins of WNT family are involved in the activation of cardiac fibrotic pathologies (150–152). Działo et al. confirmed that WNT3a-rich exosomes could specifically activate WNT/β-catenin signaling pathway and promoted fibrogenesis in post-infarcted hearts, whereas WNT5a-rich exosomes only activated non-canonical WNT pathways and induced production of profibrotic IL-6 (145). Summarizing, exosomes containing WNT proteins can regulate cardiac fibrosis *via* canonical and non-canonical WNT pathways and provide a novel strategy to treat cardiac fibrosis. The upregulated decorin and downregulated periostin in cardiomyocyte-derived exosomes had been confirmed to regulate cardiac fibrosis through targeting Ang II. Additionally, upregulated human antigen R (HuR) in macrophages significantly increased inflammatory and profibrogenic responses in fibroblast and cardiac fibrosis, suggesting that HuR might be targeted to alleviate macrophage dysfunction and pathological fibrosis (144).

### Exosomes Act as Biomarkers in Cardiac Fibrosis

Recently, researches have been devoted to using miRNAs or other molecules in serum or plasma as diagnostic or prognostic biomarkers in cardiovascular diseases. Exosomes, as the carrier of those molecular constituents, are highly associated with concurrent physiological or pathological condition. It has been shown that the level of plasma exosomal miR-425 and miR-744 was decreased while the level of miR-21 was increased during the development of heart failure, which makes them novel biomarkers for heart failure and represent the conditions of the CF (136). In addition, surface HSP70 expression in serum exosomes was obviously decreased during senescence in the model of cardiac fibrosis, while HSP70 overexpression attenuated these effects, making it a new biomarker in aging-related cardiac fibrosis (142). Therefore, exosomes may act as a promising diagnostic biomarker in cardiac fibrosis.

## Conclusion

Cardiac fibrosis, a common pathophysiologic event in most heart disease, can lead to poor tissue compliance, hardening of myocardium, and worsening of cardiac dysfunction. CFs, a major cell type of adult myocardium, play a vital role in the process of cardiac fibrosis. MCs, macrophages/monocytes, endothelial cells, and cardiomyocytes, in addition to CFs, also have a role in the fibrotic response through fibrogenic growth factors, the aldosterone/angiotensin axis, or inflammatory signals. Thus, cardiac fibrosis is a complex process involving multiple cells and regulated by multiple molecular pathways. Based on this, exosomes derived from various cell types are rich in a variety of miRNAs and proteins and could participate in intercellular communication to mediate cardiac fibrosis process, thus providing a novel strategy for the prediction and treatment of cardiac fibrosis.

## Author Contributions

WJ and YX determined the topic, wrote the initial draft, and revised the manuscript according to the reviewers' comments. XL searched the related literatures and supplemented the content of the manuscript. YY supervised the planning and execution of the research activity. All authors have given approval to the final version of the manuscript, responsible for the accuracy, and authenticity of the article.

## Conflict of Interest

The authors declare that the research was conducted in the absence of any commercial or financial relationships that could be construed as a potential conflict of interest.

## Publisher's Note

All claims expressed in this article are solely those of the authors and do not necessarily represent those of their affiliated organizations, or those of the publisher, the editors and the reviewers. Any product that may be evaluated in this article, or claim that may be made by its manufacturer, is not guaranteed or endorsed by the publisher.

## Abbreviations

ECM: extracellular matrix
CF: cardiac fibroblast
AMI: acute myocardial infarction
EMT: epithelial–mesenchymal transition
MMP: matrix metalloproteinases
DDR2: discoidin domain receptor 2
FSP1: fibroblast-specific protein 1
S100a4: S100 calcium-binding protein A4
Thy1: thymus cell antigen 1
TCF21: the transcription factor 21
PDGFR α: platelet-derived growth factor receptor α
α-SMA: α-smooth muscle actin
ED-A: extradomain A
ROS: reactive oxygen species
MC: mast cells
DAMPs: damage-associated molecular patterns
TNF: tumor necrosis factor
IL: interleukin
MCG: mast cell granule
VEGF: vascular endothelial growth factor
MSC: mesenchymal stem cell
ET: endothelin
EndMT: endothelial to mesenchymal transition
HIF: hypoxia-inducible factor
IP: interferon-γ-inducible protein
TβRII: TGF-β receptor II
IGF: insulin-like growth factor
AT2: angiotensin II type 2 receptor
PAI: plasminogen activator inhibitor
PDGF: platelet-derived growth factor
RAAS: renin–angiotensin–aldosterone system
Ang II: angiotensin II
ACE: angiotensin-converting enzyme
AT 1: angiotensin type 1
AR: adrenergic receptor
CCN2: cellular communication network factor 2
GRK2: G protein-coupled receptor kinase 2
MRTF: myocardin-related transcription factor
TSP-1: thrombospondin 1
STAT: signal transducer and activator of transcription
JAK: Janus kinase
mIL-6R: membrane-bound IL-6R
sIL-6R: soluble IL-6R
RAS: renin–angiotensin system
HIMF: hypoxia-induced mitogenic factor
TN-C: Tenascin-C
EE: early endosome
LE: late endosome
ILV: intraluminal vesicle
MVB: multivesicular body
TSG101: tumor susceptibility gene 101
MHC: major histocompatibility complex
PDCD6IP: programmed cell death 6-interacting protein
HSP: heat shock protein
SORBS2: orbin and SH3 domain-containing protein 2
PDLIM5: PDZ and LIM domain
Nrf2: nuclear factor erythroid 2-related factor 2
FoxO: forkhead transcription factors of the O class
YBX-1: Y box binding protein 1
Smurf1: SMAD-specific E3 ubiquitin protein ligase 1
HDAC4: histone deacetylase 4
MKK6: mitogen-activated protein kinase kinase 6
Dyrk2: dual-specificity tyrosine phosphorylation-regulated kinase 2
PTEN: phosphatase and tensin homolog
Mecp2: methyl CpG binding protein 2
BMP2: bone morphogenetic protein 2
EGR1: early growth response factor 1
HuR: human antigen R
Ang: angiotensin.

## References

[1] Kong P Christia P Frangogiannis NG. The pathogenesis of cardiac fibrosis. Cell Mol Life Sci. (2014) 71:549–74. 10.1007/s00018-013-1349-6 23649149 PMC3769482

[2] Berk BC Fujiwara K Lehoux S. ECM remodeling in hypertensive heart disease. J Clin Invest. (2007) 117:568–75. 10.1172/JCI31044 17332884 PMC1804378

[3] Frangogiannis NG. Regulation of the inflammatory response in cardiac repair. Circ Res. (2012) 110:159–73. 10.1161/CIRCRESAHA.111.243162 22223212 PMC3690135

[4] Tian J An X Niu L. Myocardial fibrosis in congenital and pediatric heart disease. Exp Ther Med. (2017) 13:1660–4. 10.3892/etm.2017.4224 28565750 PMC5443200

[5] Ytrehus K Hulot JS Perrino C Schiattarella GG Madonna R. Perivascular fibrosis and the microvasculature of the heart. Still hidden secrets of pathophysiology? Vascu Pharmacol. (2018) 107:78–83. 10.1016/j.vph.2018.04.007 29709645

[6] Baudino TA Carver W Giles W Borg TK. Cardiac fibroblasts: friend or foe? Am J Physiol Heart Circul Physiol. (2006) 291:H1015–26. 10.1152/ajpheart.00023.2006 16617141

[7] Cleutjens JP Verluyten MJ Smiths JF Daemen MJ. Collagen remodeling after myocardial infarction in the rat heart. Am J Pathol. (1995) 147:325–38. 7639329 PMC1869816

[8] Cohn JN Ferrari R Sharpe N. Cardiac remodeling–concepts and clinical implications: a consensus paper from an international forum on cardiac remodeling. Behalf of an international forum on cardiac remodeling. J Am Coll Cardiol. (2000) 35:569–82. 10.1016/S0735-1097(99)00630-0 10716457

[9] Corrado C Raimondo S Chiesi A Ciccia F De Leo G Alessandro R. Exosomes as intercellular signaling organelles involved in health and disease: basic science and clinical applications. Int J Mol Sci. (2013) 14:5338–66. 10.3390/ijms14035338 23466882 PMC3634447

[10] Cerezo-Magaña M Bång-Rudenstam A Belting M. The pleiotropic role of proteoglycans in extracellular vesicle mediated communication in the tumor microenvironment. Semin Cancer Biol. (2020) 62:99–107. 10.1016/j.semcancer.2019.07.001 31276785

[11] Bang C Batkai S Dangwal S Gupta SK Foinquinos A Holzmann A. Cardiac fibroblast-derived microRNA passenger strand-enriched exosomes mediate cardiomyocyte hypertrophy. J Clin Invest. (2014) 124:2136–46. 10.1172/JCI70577 24743145 PMC4001534

[12] Wang C Zhang C Liu L A X Chen B Li Y. Macrophage-Derived mir-155-Containing exosomes suppress fibroblast proliferation and promote fibroblast inflammation during cardiac injury. Mol Ther. (2017) 25:192–204. 10.1016/j.ymthe.2016.09.001 28129114 PMC5363311

[13] Vogel W Gish GD Alves F Pawson T. The discoidin domain receptor tyrosine kinases are activated by collagen. Mol Cell. (1997) 1:13–23. 10.1016/S1097-2765(00)80003-9 9659899

[14] Souders CA Bowers SL Baudino TA. Cardiac fibroblast: the renaissance cell. Circ Res. (2009) 105:1164–76. 10.1161/CIRCRESAHA.109.209809 19959782 PMC3345531

[15] Morales MO Price RL Goldsmith EC. Expression of discoidin domain receptor 2 (DDR2) in the developing heart. Microsc Microanal. (2005) 11:260–7. 10.1017/S1431927605050518 16060979

[16] Fan D Takawale A Lee J Kassiri Z. Cardiac fibroblasts, fibrosis and extracellular matrix remodeling in heart disease. Fibrogenesis Tissue Repair. (2012) 5:15. 10.1186/1755-1536-5-15 22943504 PMC3464725

[17] Goldsmith EC Hoffman A Morales MO Potts JD Price RL McFadden A. Organization of fibroblasts in the heart. Dev Dyn. (2004) 230:787–94. 10.1002/dvdy.20095 15254913

[18] Banerjee I Fuseler JW Price RL Borg TK Baudino TA. Determination of cell types and numbers during cardiac development in the neonatal and adult rat and mouse. Am J Physiol Heart Circ Physiol. (2007) 293:H1883–91. 10.1152/ajpheart.00514.2007 17604329

[19] Lane EB Hogan BL Kurkinen M Garrels JI. Co-expression of vimentin and cytokeratins in parietal endoderm cells of early mouse embryo. Nature. (1983) 303:701–4. 10.1038/303701a0 6190091

[20] Ali SR Ranjbarvaziri S Talkhabi M Zhao P Subat A Hojjat A. Developmental heterogeneity of cardiac fibroblasts does not predict pathological proliferation and activation. Circ Res. (2014) 115:625–35. 10.1161/CIRCRESAHA.115.303794 25037571

[21] Franke WW Schmid E Osborn M Weber K. Intermediate-sized filaments of human endothelial cells. J Cell Biol. (1979) 81:570–80. 10.1083/jcb.81.3.570 379021 PMC2110384

[22] Moore-Morris T Guimarães-Camboa N Yutzey KE Pucéat M Evans SM. Cardiac fibroblasts: from development to heart failure. J Mol Med. (2015) 93:823–30. 10.1007/s00109-015-1314-y 26169532 PMC4512919

[23] Strutz F Okada H Lo CW Danoff T Carone RL Tomaszewski JE. Identification and characterization of a fibroblast marker: FSP1. J Cell Biol. (1995) 130:393–405. 10.1083/jcb.130.2.393 7615639 PMC2199940

[24] Doppler SA Carvalho C Lahm H Deutsch MA Dreßen M Puluca N. Cardiac fibroblasts: more than mechanical support. J Thorac Dis. (2017) 9 (Suppl 1):S36–51. 10.21037/jtd.2017.03.122 28446967 PMC5383558

[25] Acharya A Baek ST Huang G Eskiocak B Goetsch S Sung CY. The bHLH transcription factor Tcf21 is required for lineage-specific EMT of cardiac fibroblast progenitors. Development. (2012) 139:2139–49. 10.1242/dev.079970 22573622 PMC3357908

[26] Crisan M Yap S Casteilla L Chen CW Corselli M Park TS. A perivascular origin for mesenchymal stem cells in multiple human organs. Cell Stem Cell. (2008) 3:301–13. 10.1016/j.stem.2008.07.003 18786417

[27] Jurisic G Iolyeva M Proulx ST Halin C Detmar M. Thymus cell antigen 1 (Thy1, CD90) is expressed by lymphatic vessels and mediates cell adhesion to lymphatic endothelium. Exp Cell Res. (2010) 316:2982–92. 10.1016/j.yexcr.2010.06.013 20599951 PMC3398154

[28] Braitsch CM Kanisicak O van Berlo JH Molkentin JD Yutzey KE. Differential expression of embryonic epicardial progenitor markers and localization of cardiac fibrosis in adult ischemic injury and hypertensive heart disease. J Mol Cell Cardiol. (2013) 65:108–19. 10.1016/j.yjmcc.2013.10.005 24140724 PMC3848425

[29] Ivey MJ Tallquist MD. Defining the cardiac fibroblast. Circ J. (2016) 80:2269–76. 10.1253/circj.CJ-16-1003 27746422 PMC5588900

[30] Chong JJ Reinecke H Iwata M Torok-Storb B Stempien-Otero A Murry CE. Progenitor cells identified by PDGFR-alpha expression in the developing and diseased human heart. Stem Cells Dev. (2013) 22:1932–43. 10.1089/scd.2012.0542 23391309 PMC3685392

[31] Cuttler AS LeClair RJ Stohn JP Wang Q Sorenson CM Liaw L. Characterization of Pdgfrb-Cre transgenic mice reveals reduction of ROSA26 reporter activity in remodeling arteries. Genesis. (2011) 49:673–80. 10.1002/dvg.20769 21557454 PMC3244048

[32] Yata Y Scanga A Gillan A Yang L Reif S Breindl M. DNase I-hypersensitive sites enhance alpha1(I) collagen gene expression in hepatic stellate cells. Hepatology. (2003) 37:267–76. 10.1053/jhep.2003.50067 12540776

[33] Porter KE Turner NA. Cardiac fibroblasts: at the heart of myocardial remodeling. Pharmacol Ther. (2009) 123:255–78. 10.1016/j.pharmthera.2009.05.002 19460403

[34] Zeisberg EM Tarnavski O Zeisberg M Dorfman AL McMullen JR Gustafsson E. Endothelial-to-mesenchymal transition contributes to cardiac fibrosis. Nat Med. (2007) 13:952–61. 10.1038/nm1613 17660828

[35] Katsuragi N Morishita R Nakamura N Ochiai T Taniyama Y Hasegawa Y. Periostin as a novel factor responsible for ventricular dilation. Circulation. (2004) 110:1806–13. 10.1161/01.CIR.0000142607.33398.54 15381649

[36] Kudo A. Periostin in fibrillogenesis for tissue regeneration: periostin actions inside and outside the cell. Cell Mol Life Sci. (2011) 68:3201–7. 10.1007/s00018-011-0784-5 21833583 PMC3173633

[37] Norris RA Borg TK Butcher JT Baudino TA Banerjee I Markwald RR. Neonatal and adult cardiovascular pathophysiological remodeling and repair: developmental role of periostin. Ann N Y Acad Sci. (2008) 1123:30–40. 10.1196/annals.1420.005 18375575

[38] Gittenberger-de GA Vrancken PM Mentink MM Gourdie RG Poelmann RE. Epicardium-derived cells contribute a novel population to the myocardial wall and the atrioventricular cushions. Circ Res. (1998) 82:1043–52. 10.1161/01.RES.82.10.1043 9622157

[39] Muñoz-Chápuli R Pérez-Pomares JM Macías D García-Garrido L Carmona R González-Iriarte M. The epicardium as a source of mesenchyme for the developing heart. Ital J Anat Embryol. (2001) 10 6(2 Suppl. 1):187–96. 11729954

[40] Wessels A van den Hoff MJ Adamo RF Phelps AL Lockhart MM Sauls K. Epicardially derived fibroblasts preferentially contribute to the parietal leaflets of the atrioventricular valves in the murine heart. Dev Biol. (2012) 366:111–24. 10.1016/j.ydbio.2012.04.020 22546693 PMC3358438

[41] Moore-Morris T Guimarães-Camboa N Banerjee I Zambon AC Kisseleva T Velayoudon A. Resident fibroblast lineages mediate pressure overload-induced cardiac fibrosis. J Clin Invest. (2014) 124:2921–34. 10.1172/JCI74783 24937432 PMC4071409

[42] Baum J Duffy HS. Fibroblasts and myofibroblasts: what are we talking about? J Cardiovasc Pharmacol. (2011) 57:376–9. 10.1097/FJC.0b013e3182116e39 21297493 PMC3077448

[43] Hinz B Phan SH Thannickal VJ Galli A Bochaton-Piallat ML Gabbiani G. The myofibroblast: one function, multiple origins. Am J Pathol. (2007) 170:1807–16. 10.2353/ajpath.2007.070112 17525249 PMC1899462

[44] Honold L Nahrendorf M. Resident and monocyte-derived macrophages in cardiovascular disease. Circ Res. (2018) 122:113–27. 10.1161/CIRCRESAHA.117.311071 29301844 PMC5777215

[45] Chen B Frangogiannis NG. Immune cells in repair of the infarcted myocardium. Microcirculation. (2017) 24:e12305. 10.1111/micc.12305 27542099

[46] Szardien S Nef HM Troidl C Willmer M Voss S Liebetrau C. Bone marrow-derived cells contribute to cell turnover in aging murine hearts. Int J Mol Med. (2012) 30:283–7. 10.3892/ijmm.2012.995 22580818

[47] Bucala R Spiegel LA Chesney J Hogan M Cerami A. Circulating fibrocytes define a new leukocyte subpopulation that mediates tissue repair. Mol Med. (1994) 1:71–81. 10.1007/BF03403533 8790603 PMC2229929

[48] Saarinen J Kalkkinen N Welgus HG Kovanen PT. Activation of human interstitial procollagenase through direct cleavage of the Leu83-Thr84 bond by mast cell chymase. J Biol Chem. (1994) 269:18134–40. 10.1016/S0021-9258(17)32427-4 8027075

[49] Cho SH Lee SH Kato A Takabayashi T Kulka M Shin SC. Cross-talk between human mast cells and bronchial epithelial cells in plasminogen activator inhibitor-1 production *via* transforming growth factor-β1. Am J Respir Cell Mol Biol. (2015) 52:88–95. 10.1165/rcmb.2013-0399OC 24987792 PMC4370249

[50] Takai S Jin D Sakaguchi M Katayama S Muramatsu M Sakaguchi M. A novel chymase inhibitor, 4-[1-([bis-(4-methyl-phenyl)-methyl]-carbamoyl)3-(2-ethoxy-benzyl)-4-oxo-azetidine-2-yloxy]-benzoic acid (BCEAB), suppressed cardiac fibrosis in cardiomyopathic hamsters. J Pharmacol Exp Ther. (2003) 305:17–23. 10.1124/jpet.102.045179 12649348

[51] Prabhu SD Frangogiannis NG. The biological basis for cardiac repair after myocardial infarction: from inflammation to fibrosis. Circ Res. (2016) 119:91–112. 10.1161/CIRCRESAHA.116.303577 27340270 PMC4922528

[52] Wernersson S Pejler G. Mast cell secretory granules: armed for battle. Nat Rev Immunol. (2014) 14:478–94. 10.1038/nri3690 24903914

[53] Mukai K Tsai M Saito H Galli SJ. Mast cells as sources of cytokines, chemokines, and growth factors. Immunol Rev. (2018) 282:121–50. 10.1111/imr.12634 29431212 PMC5813811

[54] Wang Y Li Y Wu Y Jia L Wang J Xie B. 5TNF-α and IL-1β neutralization ameliorates angiotensin II-induced cardiac damage in male mice. Endocrinology. (2014) 155:2677–87. 10.1210/en.2013-2065 24877626

[55] Lin TJ Befus AD. Differential regulation of mast cell function by IL-10 and stem cell factor. J Immunol. (1997) 159:4015–23. 9378991

[56] Verma SK Garikipati V Krishnamurthy P Schumacher SM Grisanti LA Cimini M. Interleukin-10 inhibits bone marrow fibroblast progenitor cell-mediated cardiac fibrosis in pressure-overloaded myocardium. Circulation. (2017) 136:940–53. 10.1161/CIRCULATIONAHA.117.027889 28667100 PMC5736130

[57] Verma SK Krishnamurthy P Barefield D Singh N Gupta R Lambers E. Interleukin-10 treatment attenuates pressure overload-induced hypertrophic remodeling and improves heart function *via* signal transducers and activators of transcription 3-dependent inhibition of nuclear factor-κB. Circulation. (2012) 126:418–29. 10.1161/CIRCULATIONAHA.112.112185 22705886 PMC3422741

[58] Krishnamurthy P Rajasingh J Lambers E Qin G Losordo DW Kishore R. IL-10 inhibits inflammation and attenuates left ventricular remodeling after myocardial infarction *via* activation of STAT3 and suppression of HuR. Circ Res. (2009) 104:e9–18. 10.1161/CIRCRESAHA.108.188243 19096025 PMC2774810

[59] Nako H Kataoka K Koibuchi N Dong YF Toyama K Yamamoto E. Novel mechanism of angiotensin II-induced cardiac injury in hypertensive rats: the critical role of ASK1 and VEGF. Hypertens Res. (2012) 35:194–200. 10.1038/hr.2011.175 22089532

[60] Tang JM Luo B Xiao JH Lv YX Li XL Zhao JH. VEGF-A promotes cardiac stem cell engraftment and myocardial repair in the infarcted heart. Int J Cardiol. (2015) 183:221–31. 10.1016/j.ijcard.2015.01.050 25679991

[61] Yang L Kwon J Popov Y Gajdos GB Ordog T Brekken RA. Vascular endothelial growth factor promotes fibrosis resolution and repair in mice. Gastroenterology. (2014) 146:1339–50.e1. 10.1053/j.gastro.2014.01.061 24503129 PMC4001704

[62] Nazari M Ni NC Lüdke A Li SH Guo J Weisel RD. Mast cells promote proliferation and migration and inhibit differentiation of mesenchymal stem cells through PDGF. J Mol Cell Cardiol. (2016) 94:32–42. 10.1016/j.yjmcc.2016.03.007 26996757

[63] Xia Y Lee K Li N Corbett D Mendoza L Frangogiannis NG. Characterization of the inflammatory and fibrotic response in a mouse model of cardiac pressure overload. Histochem Cell Biol. (2009) 131:471–81. 10.1007/s00418-008-0541-5 19030868 PMC2782393

[64] Adiarto S Heiden S Vignon-Zellweger N Nakayama K Yagi K Yanagisawa M. ET-1 from endothelial cells is required for complete angiotensin II-induced cardiac fibrosis and hypertrophy. Life Sci. (2012) 91:651–7. 10.1016/j.lfs.2012.02.006 22365964

[65] Widyantoro B Emoto N Nakayama K Anggrahini DW Adiarto S Iwasa N. Endothelial cell-derived endothelin-1 promotes cardiac fibrosis in diabetic hearts through stimulation of endothelial-to-mesenchymal transition. Circulation. (2010) 121:2407–18. 10.1161/CIRCULATIONAHA.110.938217 20497976

[66] Salvador AM Nevers T Velázquez F Aronovitz M Wang B Abadía MA. Intercellular adhesion molecule 1 regulates left ventricular leukocyte infiltration, cardiac remodeling, and function in pressure overload-induced heart failure. J Am Heart Assoc. (2016) 5:e003126. 10.1161/JAHA.115.003126 27068635 PMC4943280

[67] Wei H Bedja D Koitabashi N Xing D Chen J Fox-Talbot K. Endothelial expression of hypoxia-inducible factor 1 protects the murine heart and aorta from pressure overload by suppression of TGF-β signaling. Proc Natl Acad Sci USA. (2012) 109:E841–50. 10.1073/pnas.1202081109 22403061 PMC3325701

[68] Frangogiannis NG Mendoza LH Lewallen M Michael LH Smith CW Entman ML. Induction and suppression of interferon-inducible protein 10 in reperfused myocardial infarcts may regulate angiogenesis. FASEB J. (2001) 15:1428–30. 10.1096/fj.00-0745fje 11387246

[69] Rickard AJ Morgan J Bienvenu LA Fletcher EK Cranston GA Shen JZ. Cardiomyocyte mineralocorticoid receptors are essential for deoxycorticosterone/salt-mediated inflammation and cardiac fibrosis. Hypertension. (2012) 60:1443–50. 10.1161/HYPERTENSIONAHA.112.203158 23108646

[70] Koitabashi N Danner T Zaiman AL Pinto YM Rowell J Mankowski J. Pivotal role of cardiomyocyte TGF-β signaling in the murine pathological response to sustained pressure overload. J Clin Invest. (2011) 121:2301–12. 10.1172/JCI44824 21537080 PMC3104748

[71] Ock S Lee WS Ahn J Kim HM Kang H Kim HS. Deletion of IGF-1 receptors in cardiomyocytes attenuates cardiac aging in male mice. Endocrinology. (2016) 157:336–45. 10.1210/en.2015-1709 26469138 PMC4701888

[72] Kurisu S Ozono R Oshima T Kambe M Ishida T Sugino H. Cardiac angiotensin II type 2 receptor activates the kinin/NO system and inhibits fibrosis. Hypertension. (2003) 41:99–107. 10.1161/01.HYP.0000050101.90932.14 12511537

[73] Flevaris P Khan SS Eren M Schuldt A Shah SJ Lee DC. Plasminogen activator inhibitor type i controls cardiomyocyte transforming growth factor-β and cardiac fibrosis. Circulation. (2017) 136:664–79. 10.1161/CIRCULATIONAHA.117.028145 28588076 PMC5784400

[74] Teufel A Becker D Weber SN Dooley S Breitkopf-Heinlein K Maass T. Identification of RARRES1 as a core regulator in liver fibrosis. J Mol Med. (2012) 90:1439–47. 10.1007/s00109-012-0919-7 22669512

[75] Frangogiannis NG. Chemokines in the ischemic myocardium: from inflammation to fibrosis. Inflamm Res. (2004) 53:585–95. 10.1007/s00011-004-1298-5 15693606

[76] Weber KT Sun Y Bhattacharya SK Ahokas RA Gerling IC. Myofibroblast-mediated mechanisms of pathological remodelling of the heart. Nat Rev Cardiol. (2013) 10:15–26. 10.1038/nrcardio.2012.158 23207731

[77] Hokimoto S Yasue H Fujimoto K Yamamoto H Nakao K Kaikita K. Expression of angiotensin-converting enzyme in remaining viable myocytes of human ventricles after myocardial infarction. Circulation. (1996) 94:1513–8. 10.1161/01.CIR.94.7.1513 8840838

[78] Crabos M Roth M Hahn AW Erne P. Characterization of angiotensin II receptors in cultured adult rat cardiac fibroblasts. Coupling to signaling systems and gene expression. J Clin Invest. (1994) 93:2372–8. 10.1172/JCI117243 8200970 PMC294443

[79] Schorb W Booz GW Dostal DE Conrad KM Chang KC Baker KM. Angiotensin II is mitogenic in neonatal rat cardiac fibroblasts. Circ Res. (1993) 72:1245–54. 10.1161/01.RES.72.6.1245 8495553

[80] Sadoshima J Izumo S. Molecular characterization of angiotensin II–induced hypertrophy of cardiac myocytes and hyperplasia of cardiac fibroblasts. Critical role of the AT1 receptor subtype. Circ Res. (1993) 73:413–23. 10.1161/01.RES.73.3.413 8348686

[81] Campbell SE Katwa LC. Angiotensin II stimulated expression of transforming growth factor-beta1 in cardiac fibroblasts and myofibroblasts. J Mol Cell Cardiol. (1997) 29:1947–58. 10.1006/jmcc.1997.0435 9236148

[82] Schultz JJ Witt SA Glascock BJ Nieman ML Reiser PJ Nix SL. TGF-beta1 mediates the hypertrophic cardiomyocyte growth induced by angiotensin II. J Clin Invest. (2002) 109:787–96. 10.1172/JCI0214190 11901187 PMC150912

[83] Chen S Evans T Mukherjee K Karmazyn M Chakrabarti S. Diabetes-induced myocardial structural changes: role of endothelin-1 and its receptors. J Mol Cell Cardiol. (2000) 32:1621–9. 10.1006/jmcc.2000.1197 10966825

[84] Dashwood MR Abraham D. Endothelin: from bench to bedside and back. Pharmacol Res. (2011) 63:445–7. 10.1016/j.phrs.2011.04.005 21641525

[85] Singh AD Amit S Kumar OS Rajan M Mukesh N. Cardioprotective effects of bosentan, a mixed endothelin type A and B receptor antagonist, during myocardial ischaemia and reperfusion in rats. Basic Clin Pharmacol Toxicol. (2006) 98:604–10. 10.1111/j.1742-7843.2006.pto_405.x 16700825

[86] Schiller M Javelaud D Mauviel A. TGF-beta-induced SMAD signaling and gene regulation: consequences for extracellular matrix remodeling and wound healing. J Dermatol Sci. (2004) 35:83–92. 10.1016/j.jdermsci.2003.12.006 15265520

[87] Kapur NK Wilson S Yunis AA Qiao X Mackey E Paruchuri V. Reduced endoglin activity limits cardiac fibrosis and improves survival in heart failure. Circulation. (2012) 125:2728–38. 10.1161/CIRCULATIONAHA.111.080002 22592898 PMC4774533

[88] Zhao XY Zhao LY Zheng QS Su JL Guan H Shang FJ. Chymase induces profibrotic response *via* transforming growth factor-beta 1/Smad activation in rat cardiac fibroblasts. Mol Cell Biochem. (2008) 310:159–66. 10.1007/s11010-007-9676-2 18057996

[89] Dobaczewski M Gonzalez-Quesada C Frangogiannis NG. The extracellular matrix as a modulator of the inflammatory and reparative response following myocardial infarction. J Mol Cell Cardiol. (2010) 48:504–11. 10.1016/j.yjmcc.2009.07.015 19631653 PMC2824059

[90] Gallini R Lindblom P Bondjers C Betsholtz C Andrae J. PDGF-A and PDGF-B induces cardiac fibrosis in transgenic mice. Exp Cell Res. (2016) 349:282–90. 10.1016/j.yexcr.2016.10.022 27816607

[91] Pontén A Li X Thorén P Aase K Sjöblom T Ostman A. Transgenic overexpression of platelet-derived growth factor-C in the mouse heart induces cardiac fibrosis, hypertrophy, and dilated cardiomyopathy. Am J Pathol. (2003) 163:673–82. 10.1016/S0002-9440(10)63694-2 12875986 PMC1868211

[92] Pontén A Folestad EB Pietras K Eriksson U. Platelet-derived growth factor D induces cardiac fibrosis and proliferation of vascular smooth muscle cells in heart-specific transgenic mice. Circ Res. (2005) 97:1036–45. 10.1161/01.RES.0000190590.31545.d4 16224065

[93] Chen Y Surinkaew S Naud P Qi XY Gillis MA Shi YF. JAK-STAT signalling and the atrial fibrillation promoting fibrotic substrate. Cardiovasc Res. (2017) 113:310–20. 10.1093/cvr/cvx004 28158495 PMC5852635

[94] Leask A. Potential therapeutic targets for cardiac fibrosis: TGFbeta, angiotensin, endothelin, CCN2, and PDGF, partners in fibroblast activation. Circ Res. (2010) 106:1675–80. 10.1161/CIRCRESAHA.110.217737 20538689

[95] Liao CH Akazawa H Tamagawa M Ito K Yasuda N Kudo Y. Cardiac mast cells cause atrial fibrillation through PDGF-A-mediated fibrosis in pressure-overloaded mouse hearts. J Clin Invest. (2010) 120:242–53. 10.1172/JCI39942 20038802 PMC2798688

[96] Horiuchi T Mitoma H Harashima S Tsukamoto H Shimoda T. Transmembrane TNF-alpha: structure, function and interaction with anti-TNF agents. Rheumatology. (2010) 49:1215–28. 10.1093/rheumatology/keq031 20194223 PMC2886310

[97] Hamid T Gu Y Ortines RV Bhattacharya C Wang G Xuan YT. Divergent tumor necrosis factor receptor-related remodeling responses in heart failure: role of nuclear factor-kappaB and inflammatory activation. Circulation. (2009) 119:1386–97. 10.1161/CIRCULATIONAHA.108.802918 19255345 PMC2730645

[98] Frangogiannis NG. Cardiac fibrosis: cell biological mechanisms, molecular pathways and therapeutic opportunities. Mol Aspects Med. (2019) 65:70–99. 10.1016/j.mam.2018.07.001 30056242

[99] Valiente-Alandi I Potter SJ Salvador AM Schafer AE Schips T Carrillo-Salinas F. Inhibiting fibronectin attenuates fibrosis and improves cardiac function in a model of heart failure. Circulation. (2018) 138:1236–52. 10.1161/CIRCULATIONAHA.118.034609 29653926 PMC6186194

[100] Mihara M Hashizume M Yoshida H Suzuki M Shiina M. IL-6/IL-6 receptor system and its role in physiological and pathological conditions. Clin Sci. (2012) 122:143–59. 10.1042/CS20110340 22029668

[101] Choy EH De Benedetti F Takeuchi T Hashizume M John MR Kishimoto T. Translating IL-6 biology into effective treatments. Nat Rev Rheumatol. (2020) 16:335–45. 10.1038/s41584-020-0419-z 32327746 PMC7178926

[102] Chou CH Hung CS Liao CW Wei LH Chen CW Shun CT. IL-6 trans-signalling contributes to aldosterone-induced cardiac fibrosis. Cardiovasc Res. (2018) 114:690–702. 10.1093/cvr/cvy013 29360942

[103] Eid RA Alkhateeb MA El-Kott AF Eleawa SM Zaki M Alaboodi SA. A high-fat diet rich in corn oil induces cardiac fibrosis in rats by activating JAK2/STAT3 and subsequent activation of ANG II/TGF-1β/Smad3 pathway: the role of ROS and IL-6 trans-signaling. J Food Biochem. (2019) 43:e12952. 10.1111/jfbc.12952 31368573

[104] Kumar S Wang G Zheng N Cheng W Ouyang K Lin H. HIMF (hypoxia-induced mitogenic factor)-IL (Interleukin)-6 signaling mediates cardiomyocyte-fibroblast crosstalk to promote cardiac hypertrophy and fibrosis. Hypertension. (2019) 73:1058–70. 10.1161/HYPERTENSIONAHA.118.12267 30827145

[105] Shimojo N Hashizume R Kanayama K Hara M Suzuki Y Nishioka T. Tenascin-C may accelerate cardiac fibrosis by activating macrophages *via* the integrin αVβ3/nuclear factor-κB/interleukin-6 axis. Hypertension. (2015) 66:757–66. 10.1161/HYPERTENSIONAHA.115.06004 26238448

[106] Travers JG Kamal FA Robbins J Yutzey KE Blaxall BC. Cardiac fibrosis: the fibroblast awakens. Circ Res. (2016) 118:1021–40. 10.1161/CIRCRESAHA.115.306565 26987915 PMC4800485

[107] Vandergriff A Huang K Shen D Hu S Hensley MT Caranasos TG. Targeting regenerative exosomes to myocardial infarction using cardiac homing peptide. Theranostics. (2018) 8:1869–78. 10.7150/thno.20524 29556361 PMC5858505

[108] Han C Zhou J Liang C Liu B Pan X Zhang Y. Human umbilical cord mesenchymal stem cell derived exosomes encapsulated in functional peptide hydrogels promote cardiac repair. Biomater Sci. (2019) 7:2920–33. 10.1039/C9BM00101H 31090763

[109] Raposo G Tenza D Mecheri S Peronet R Bonnerot C Desaymard C. Accumulation of major histocompatibility complex class II molecules in mast cell secretory granules and their release upon degranulation. Mol Biol Cell. (1997) 8:2631–45. 10.1091/mbc.8.12.2631 9398681 PMC25733

[110] Li N Rochette L Wu Y Rosenblatt-Velin N. New insights into the role of exosomes in the heart after myocardial infarction. J Cardiovasc Transl Res. (2019) 12:18–27. 10.1007/s12265-018-9831-z 30173401

[111] Barile L Moccetti T Marbán E Vassalli G. Roles of exosomes in cardioprotection. Eur Heart J. (2017) 38:1372–79. 10.1093/eurheartj/ehw304 27443883

[112] Hosseini-Beheshti E Pham S Adomat H Li N Tomlinson GE. Exosomes as biomarker enriched microvesicles: characterization of exosomal proteins derived from a panel of prostate cell lines with distinct AR phenotypes. Mol Cell Proteomics. (2012) 11:863–85. 10.1074/mcp.M111.014845 22723089 PMC3494141

[113] Skotland T Sandvig K Llorente A. Lipids in exosomes: current knowledge and the way forward. Prog Lipid Res. (2017) 66:30–41. 10.1016/j.plipres.2017.03.001 28342835

[114] Gao XF Wang ZM Wang F Gu Y Zhang JJ Chen SL. Exosomes in coronary artery disease. Int J Biol Sci. (2019) 15:2461–70. 10.7150/ijbs.36427 31595163 PMC6775305

[115] Konoshenko MY Lekchnov EA Vlassov AV Laktionov PP. Isolation of extracellular vesicles: general methodologies and latest trends. Biomed Res Int. (2018) 2018:8545347. 10.1155/2018/8545347 29662902 PMC5831698

[116] Turturici G Tinnirello R Sconzo G Geraci F. Extracellular membrane vesicles as a mechanism of cell-to-cell communication: advantages and disadvantages. Am J Physiol Cell Physiol. (2014) 306:C621–33. 10.1152/ajpcell.00228.2013 24452373

[117] Tian C Gao L Zimmerman MC Zucker IH. Myocardial infarction-induced microRNA-enriched exosomes contribute to cardiac Nrf2 dysregulation in chronic heart failure. Am J Physiol Heart Circ Physiol. (2018) 314:H928–39. 10.1152/ajpheart.00602.2017 29373037 PMC6008149

[118] Wang B Wang ZM Ji JL Gan W Zhang A Shi HJ. Macrophage-Derived exosomal Mir-155 regulating cardiomyocyte pyroptosis and hypertrophy in uremic cardiomyopathy. JACC Basic Transl Sci. (2020) 5:148–66. 10.1016/j.jacbts.2019.10.011 32140622 PMC7046511

[119] Gollmann-Tepeköylü C Pölzl L Graber M Hirsch J Nägele F Lobenwein D. miR-19a-3p containing exosomes improve function of ischaemic myocardium upon shock wave therapy. Cardiovasc Res. (2020) 116:1226–36. 10.1093/cvr/cvz209 31410448

[120] Lin F Zeng Z Song Y Li L Wu Z Zhang X. YBX-1 mediated sorting of miR-133 into hypoxia/reoxygenation-induced EPC-derived exosomes to increase fibroblast angiogenesis and MEndoT. Stem Cell Res Ther. (2019) 10:263. 10.1186/s13287-019-1377-8 31443679 PMC6708233

[121] Liu W Zhang H Mai J Chen Z Huang T Wang S. Distinct anti-fibrotic effects of exosomes derived from endothelial colony-forming cells cultured under normoxia and hypoxia. Med Sci Monit. (2018) 24:6187–99. 10.12659/MSM.911306 30183690 PMC6134891

[122] Chaturvedi P Kalani A Medina I Familtseva A Tyagi SC. Cardiosome mediated regulation of MMP9 in diabetic heart: role of mir29b and mir455 in exercise. J Cell Mol Med. (2015) 19:2153–61. 10.1111/jcmm.12589 25824442 PMC4568920

[123] Yuan J Liu H Gao W Zhang L Ye Y Yuan L. MicroRNA-378 suppresses myocardial fibrosis through a paracrine mechanism at the early stage of cardiac hypertrophy following mechanical stress. Theranostics. (2018) 8:2565–82. 10.7150/thno.22878 29721099 PMC5928909

[124] Yang J Yu X Xue F Li Y Liu W Zhang S. Exosomes derived from cardiomyocytes promote cardiac fibrosis *via* myocyte-fibroblast cross-talk. Am J Transl Res. (2018) 10:4350–66. 30662677 PMC6325490

[125] Yu B Kim HW Gong M Wang J Millard RW Wang Y. Exosomes secreted from GATA-4 overexpressing mesenchymal stem cells serve as a reservoir of anti-apoptotic microRNAs for cardioprotection. Int J Cardiol. (2015) 182:349–60. 10.1016/j.ijcard.2014.12.043 25590961 PMC4382384

[126] Zhu J Lu K Zhang N Zhao Y Ma Q Shen J. Myocardial reparative functions of exosomes from mesenchymal stem cells are enhanced by hypoxia treatment of the cells *via* transferring microRNA-210 in an nSMase2-dependent way. Artif Cells Nanomed Biotechnol. (2018) 46:1659–70. 10.1080/21691401.2017.1388249 29141446 PMC5955787

[127] Feng Y Huang W Wani M Yu X Ashraf M. Ischemic preconditioning potentiates the protective effect of stem cells through secretion of exosomes by targeting Mecp2 *via* miR-22. PLoS ONE. (2014) 9:e88685. 10.1371/journal.pone.0088685 24558412 PMC3928277

[128] Shao L Zhang Y Pan X Liu B Liang C Zhang Y. Knockout of beta-2 microglobulin enhances cardiac repair by modulating exosome imprinting and inhibiting stem cell-induced immune rejection. Cell Mol Life Sci. (2020) 77:937–52. 10.1007/s00018-019-03220-3 31312880 PMC11104803

[129] Wang B Zhang A Wang H Klein JD Tan L Wang ZM. miR-26a limits muscle wasting and cardiac fibrosis through exosome-mediated microRNA transfer in chronic kidney disease. Theranostics. (2019) 9:1864–77. 10.7150/thno.29579 31037144 PMC6485283

[130] Bier A Berenstein P Kronfeld N Morgoulis D Ziv-Av A Goldstein H. Placenta-derived mesenchymal stromal cells and their exosomes exert therapeutic effects in duchenne muscular dystrophy. Biomaterials. (2018) 174:67–78. 10.1016/j.biomaterials.2018.04.055 29783118

[131] Ibrahim A Li C Rogers R Fournier M Li L Vaturi SD. Augmenting canonical Wnt signalling in therapeutically inert cells converts them into therapeutically potent exosome factories. Nat Biomed Eng. (2019) 3:695–705. 10.1038/s41551-019-0448-6 31451800 PMC6736698

[132] Luo Q Guo D Liu G Chen G Hang M Jin M. Exosomes from MiR-126-Overexpressing adscs are therapeutic in relieving acute myocardial ischaemic injury. Cell Physiol Biochem. (2017) 44:2105–16. 10.1159/000485949 29241208

[133] Izarra A Moscoso I Levent E Cañón S Cerrada I Díez-Juan A. miR-133a enhances the protective capacity of cardiac progenitors cells after myocardial infarction. Stem Cell Rep. (2014) 3:1029–42. 10.1016/j.stemcr.2014.10.010 25465869 PMC4264058

[134] Milano G Biemmi V Lazzarini E Balbi C Ciullo A Bolis S. Intravenous administration of cardiac progenitor cell-derived exosomes protects against doxorubicin/trastuzumab-induced cardiac toxicity. Cardiovasc Res. (2020) 116:383–92. 10.1093/cvr/cvz108 31098627

[135] Pan J Alimujiang M Chen Q Shi H Luo X. Exosomes derived from miR-146a-modified adipose-derived stem cells attenuate acute myocardial infarction-induced myocardial damage *via* downregulation of early growth response factor 1. J Cell Biochem. (2019) 120:4433–43. 10.1002/jcb.27731 30362610

[136] Wang L Liu J Xu B Liu YL Liu Z. Reduced exosome miR-425 and miR-744 in the plasma represents the progression of fibrosis and heart failure. Kaohsiung J Med Sci. (2018) 34:626–33. 10.1016/j.kjms.2018.05.008 30392569 PMC11915641

[137] Kang JY Park H Kim H Mun D Park H Yun N. Human peripheral blood-derived exosomes for microRNA delivery. Int J Mol Med. (2019) 43:2319–28. 10.3892/ijmm.2019.4202 30942393 PMC6488179

[138] Cai L Chao G Li W Zhu J Li F Qi B. Activated CD4(+) T cells-derived exosomal miR-142-3p boosts post-ischemic ventricular remodeling by activating myofibroblast. Aging. (2020) 12:7380–96. 10.18632/aging.103084 32327611 PMC7202529

[139] Wang X Gu H Huang W Peng J Li Y Yang L. Hsp20-Mediated activation of exosome biogenesis in cardiomyocytes improves cardiac function and angiogenesis in diabetic mice. Diabetes. (2016) 65:3111–28. 10.2337/db15-1563 27284111 PMC5033265

[140] Datta R Bansal T Rana S Datta K Datta CR Chawla-Sarkar M. Myocyte-Derived Hsp90 modulates collagen upregulation *via* biphasic activation of STAT-3 in fibroblasts during cardiac hypertrophy. Mol Cell Biol. (2017) 37:e00611–16. 10.1128/MCB.00611-16 28031326 PMC5335508

[141] Kuo HF Hsieh CC Wang SC Chang CY Hung CH Kuo PL. Simvastatin attenuates cardiac fibrosis *via* regulation of cardiomyocyte-derived exosome secretion. J Clin Med. (2019) 8:794. 10.3390/jcm8060794 31167519 PMC6617127

[142] Yang J Yu XF Li YY Xue FT Zhang S. Decreased HSP70 expression on serum exosomes contributes to cardiac fibrosis during senescence. Eur Rev Med Pharmacol Sci. (2019) 23:3993–4001. 10.26355/eurrev_201905_17829 31115028

[143] Wang X Chen Y Zhao Z Meng Q Yu Y Sun J. Engineered exosomes with ischemic myocardium-targeting peptide for targeted therapy in myocardial infarction. J Am Heart Assoc. (2018) 7:e008737. 10.1161/JAHA.118.008737 30371236 PMC6201471

[144] Govindappa PK Patil M Garikipati V Verma SK Saheera S Narasimhan G. Targeting exosome-associated human antigen R attenuates fibrosis and inflammation in diabetic heart. FASEB J. (2020) 34:2238–51. 10.1096/fj.201901995R 31907992 PMC8286699

[145] Działo E Rudnik M Koning RI Czepiel M Tkacz K Baj-Krzyworzeka M. WNT3a and WNT5a transported by exosomes activate wnt signaling pathways in human cardiac fibroblasts. Int J Mol Sci. (2019) 20:1436. 10.3390/ijms20061436 30901906 PMC6472055

[146] Thum T Gross C Fiedler J Fischer T Kissler S Bussen M. MicroRNA-21 contributes to myocardial disease by stimulating MAP kinase signalling in fibroblasts. Nature. (2008) 456:980–4. 10.1038/nature07511 19043405

[147] Nie X Fan J Li H Yin Z Zhao Y Dai B. miR-217 promotes cardiac hypertrophy and dysfunction by targeting PTEN. Mol Ther Nucleic Acids. (2018) 12:254–66. 10.1016/j.omtn.2018.05.013 30195764 PMC6005806

[148] Richter K Haslbeck M Buchner J. The heat shock response: life on the verge of death. Mol Cell. (2010) 40:253–66. 10.1016/j.molcel.2010.10.006 20965420

[149] Fan GC Kranias EG. Small heat shock protein 20 (HspB6) in cardiac hypertrophy and failure. J Mol Cell Cardiol. (2011) 51:574–7. 10.1016/j.yjmcc.2010.09.013 20869365 PMC3033453

[150] Laeremans H Hackeng TM van Zandvoort MA Thijssen VL Janssen BJ Ottenheijm HC. Blocking of frizzled signaling with a homologous peptide fragment of wnt3a/wnt5a reduces infarct expansion and prevents the development of heart failure after myocardial infarction. Circulation. (2011) 124:1626–35. 10.1161/CIRCULATIONAHA.110.976969 21931076

[151] Blyszczuk P Müller-Edenborn B Valenta T Osto E Stellato M Behnke S. Transforming growth factor-β-dependent Wnt secretion controls myofibroblast formation and myocardial fibrosis progression in experimental autoimmune myocarditis. Eur Heart J. (2017) 38:1413–25. 10.1093/eurheartj/ehw116 27099262

[152] Abraityte A Vinge LE Askevold ET Lekva T Michelsen AE Ranheim T. Wnt5a is elevated in heart failure and affects cardiac fibroblast function. J Mol Med. (2017) 95:767–77. 10.1007/s00109-017-1529-1 28357477

